# Supplementary material for: Image-Based Annotation of Chemogenomic Libraries for Phenotypic Screening
Source: Molecules. 2022 Feb 21;27(4):1439. doi: 10.3390/molecules27041439 (PMC8878468; doi:10.3390/molecules27041439)
Supplement: Supplementary file 1 [file molecules-27-01439-s001.zip › molecules-1568774-supplementary.pdf]

Image-Based Annotation of Chemogenomic Libraries for Phenotypic Screening

Amelie Tjaden [1,2], Apirat Chaikuad [1,2], Eric Kowarz [3], Rolf Marschalek [3], Stefan Knapp [1,2], Martin Schröder [1,2,*] and Susanne Müller [1,2,*]

[1] Institute of Pharmaceutical Chemistry, Goethe University Frankfurt, Max-von-Laue-Str.9, 60438 Frankfurt, Germany; tjaden@pharmchem.uni-frankfurt.de (A.T.); chaikuad@pharmchem.uni-frankfurt.de (A.C.); knapp@pharmchem.uni-frankfurt.de (S.K.)

[2] Structural Genomics Consortium, BMLS, Goethe University Frankfurt, Max-von-Laue-Str. 15, 60438 Frankfurt, Germany

[3] Institute of Pharmaceutical Biology, Goethe University, Max-von-Laue-Str.9, 60438 Frankfurt, Germany; kowarz@em.uni-frankfurt.de (E.K.); rolf.marschalek@em.uni-frankfurt.de (R.M.)

* Correspondence: m.schroeder@pharmchem.uni-frankfurt.de (M.S.); susanne.mueller-knapp@bmls.de (S.M.)

Table of contents

Supplementary Figure S1: Hoechst33342 dye titration in HeLa cells after 20 h.

Supplementary Figure S2. Analysis of Cell Nuclei by Hoechst Channel Intensity level

Supplementary Figure S3: Fluorescence Spectrum of berzosertib

Supplementary Figure S4: Validation of Multiplex high Via protocol

Supplementary Figure S5: Hoechst High Intensity Object

Supplementary Figure S6: Viability analysis over nuclei gating protocol

Supplementary Figure S7: Phenotypical property analysis in HEK293T cells

Supplementary Figure S8: Phenotypical property analysis in MRC-9 cells

Supplementary Figure S9: Spectra Viewer visualization

Supplementary Table S1: Concentrations in µM of tested cell staining dyes

Supplementary Table S2: reference compounds tested in High-Via Extend protocol

Supplementary Table S3: Compounds tested in FUCCI Assay System

Supplementary Table S4: Additional data FUCCI assay (Excel)

Supplementary Table S5: References used for Multiplex protocol

Supplementary Table S6: Trainings set

Supplementary Table S7: Features of machine learning algorithm

Supplementary Table S8: Multiplex Chemogenomic data (Excel)

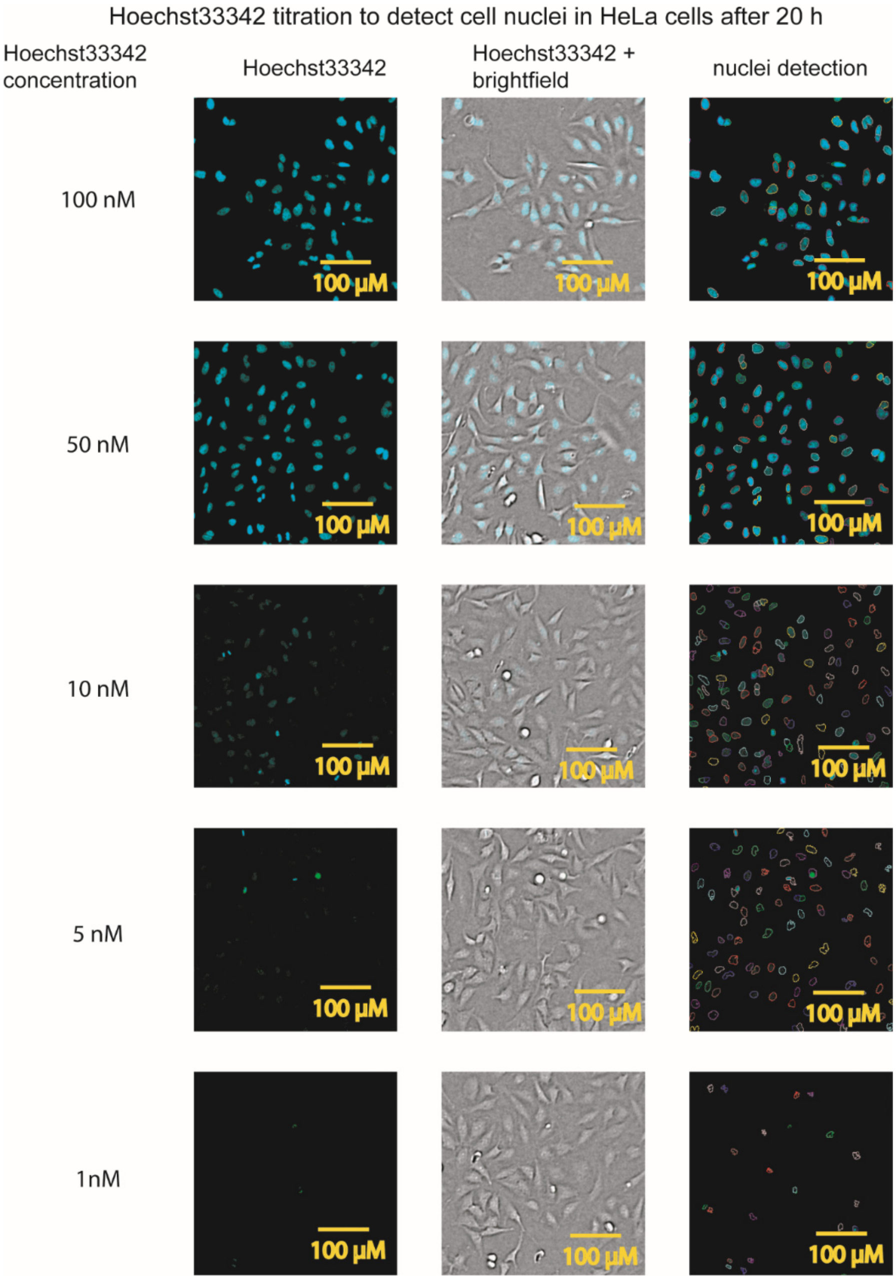

Supplementary Figure S1: Hoechst33342 dye titration in HeLa cells after 20 h. Fluorescence images, bright field images and nuclei detection of HeLa cells, after 20 h of exposure to different concentrations of Hoechst33342 stain (100 nM, 50 nM, 10 nM, 5 nM, 1 nM).

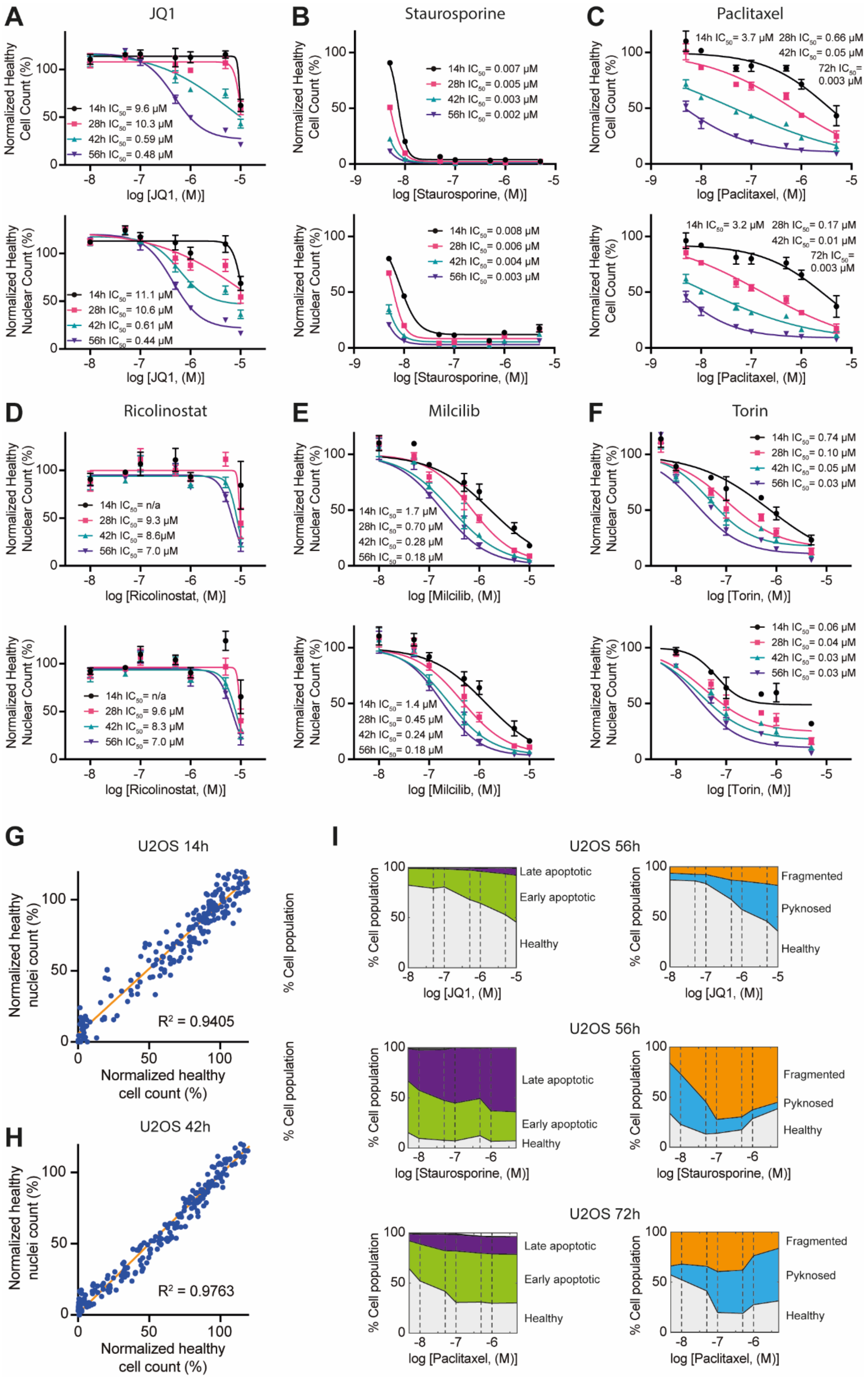
A
JQ1
Normalized Healthy Cell Count (%)
14h IC50= 9.6 µM
28h IC50= 10.3 µM
42h IC50= 0.59 µM
56h IC50= 0.48 µM
log [JQ1, (M)]
Normalized Healthy Nuclear Count (%)
14h IC50= 11.1 µM
28h IC50= 10.6 µM
42h IC50= 0.61 µM
56h IC50= 0.44 µM
log [JQ1, (M)]
B
Staurosporine
Normalized Healthy Cell Count (%)
14h IC50= 0.007 µM
28h IC50= 0.005 µM
42h IC50= 0.003 µM
56h IC50= 0.002 µM
log [Staurosporine, (M)]
Normalized Healthy Nuclear Count (%)
14h IC50= 0.008 µM
28h IC50= 0.006 µM
42h IC50= 0.004 µM
56h IC50= 0.003 µM
log [Staurosporine, (M)]
C
Paclitaxel
Normalized Healthy Cell Count (%)
14h IC50= 3.7 µM 28h IC50= 0.66 µM
42h IC50= 0.05 µM
72h IC50= 0.003 µM
log [Paclitaxel, (M)]
Normalized Healthy Nuclear Count (%)
14h IC50= 3.2 µM 28h IC50= 0.17 µM
42h IC50= 0.01 µM
72h IC50= 0.003 µM
log [Paclitaxel, (M)]
D
Ricolinostat
Normalized Healthy Nuclear Count (%)
14h IC50= n/a
28h IC50= 9.3 µM
42h IC50= 8.6µM
56h IC50= 7.0 µM
log [Ricolinostat, (M)]
14h IC50= n/a
28h IC50= 9.6 µM
42h IC50= 8.3 µM
56h IC50= 7.0 µM
log [Ricolinostat, (M)]
E
Milcilib
Normalized Healthy Nuclear Count (%)
14h IC50= 1.7 µM
28h IC50= 0.70 µM
42h IC50= 0.28 µM
56h IC50= 0.18 µM
log [Milcilib, (M)]
14h IC50= 1.4 µM
28h IC50= 0.45 µM
42h IC50= 0.24 µM
56h IC50= 0.18 µM
log [Milcilib, (M)]
F
Torin
Normalized Healthy Nuclear Count (%)
14h IC50= 0.74 µM
28h IC50= 0.10 µM
42h IC50= 0.05 µM
56h IC50= 0.03 µM
log [Torin, (M)]
14h IC50= 0.06 µM
28h IC50= 0.04 µM
42h IC50= 0.03 µM
56h IC50= 0.03 µM
log [Torin, (M)]
G
U2OS 14h
Normalized healthy nuclei count (%)
R² = 0.9405
Normalized healthy cell count (%)
H
U2OS 42h
Normalized healthy nuclei count (%)
R² = 0.9763
Normalized healthy cell count (%)
I
U2OS 56h
% Cell population
Late apoptotic
Early apoptotic
Healthy
log [JQ1, (M)]
Fragmented
Pyknosed
Healthy
log [JQ1, (M)]
U2OS 56h
Late apoptotic
Early apoptotic
Healthy
log [Staurosporine, (M)]
Fragmented
Pyknosed
Healthy
log [Staurosporine, (M)]
U2OS 72h
Late apoptotic
Early apoptotic
Healthy
log [Paclitaxel, (M)]
Fragmented
Pyknosed
Healthy
log [Paclitaxel, (M)]

Supplementary Figure S2. Analysis of Cell Nuclei by Hoechst Channel Intensity level A Normalized healthy cell count and normalized healthy nuclear count of different concentrations (0.01 µM, 0.05 µM, 0.1 µM, 0.5 µM, 1 µM, 5 µM, 10 µM) of JQ1 exposure with calculated IC50 values after 14h, 28h, 42h and 56h. **B** Normalized healthy cell count and normalized healthy nuclear count of different concentrations (0.01 µM, 0.05 µM, 0.1 µM, 0.5 µM, 1 µM, 5 µM, 10 µM) of staurosporine exposure with calculated IC50 values after 14h, 28h, 42h and 56h. **C** Normalized healthy cell count and normalized healthy nuclear count of different concentrations (0.01 µM, 0.05 µM, 0.1 µM, 0.5 µM, 1 µM, 5 µM, 10 µM) of paclitaxel exposure with calculated IC50 values after 14h, 28h, 42h and 56h. **D** Normalized healthy cell count and normalized healthy nuclear count of different concentrations (0.01 µM, 0.05 µM, 0.1 µM, 0.5 µM, 1 µM, 5 µM, 10 µM) of ricolinostat exposure with calculated IC50 values after 14h, 28h, 42h and 56h. **E** Normalized healthy cell count and normalized healthy nuclear count of different concentrations (0.01 µM, 0.05 µM, 0.1 µM, 0.5 µM, 1 µM, 5 µM, 10 µM) of milciclib exposure with calculated IC50 values after 14h, 28h, 42h and 56h. **F** Normalized healthy cell count and normalized healthy nuclear count of different concentrations (0.05 µM, 0.01 µM, 0.05 µM, 0.1 µM, 0.5 µM, 1 µM, 5 µM) of cisplatin exposure with calculated IC50 values after 14h, 28h, 42h and 56h. **G** Correlation between healthy cell count and healthy nuclei count after 14 h of compound exposure normalized to healthy cells exposed to DMSO 0,1% in U2OS cells. **H** Correlation between healthy cell count and healthy nuclei count after 42 h of compound exposure normalized to healthy cells exposed to DMSO 0,1% in U2OS cells. **I** Fractions of High Via gating and fractions of healthy, fragmented and pyknosed nuclei after exposure to different concentrations (0.01 µM, 0.05 µM, 0.1 µM, 0.5 µM, 1 µM, 5 µM, 10 µM) of JQ1, staurosporine or paclitaxel in U2OS cells after 56h (JQ1, staurosporine) or 72h (paclitaxel) of compound exposure.

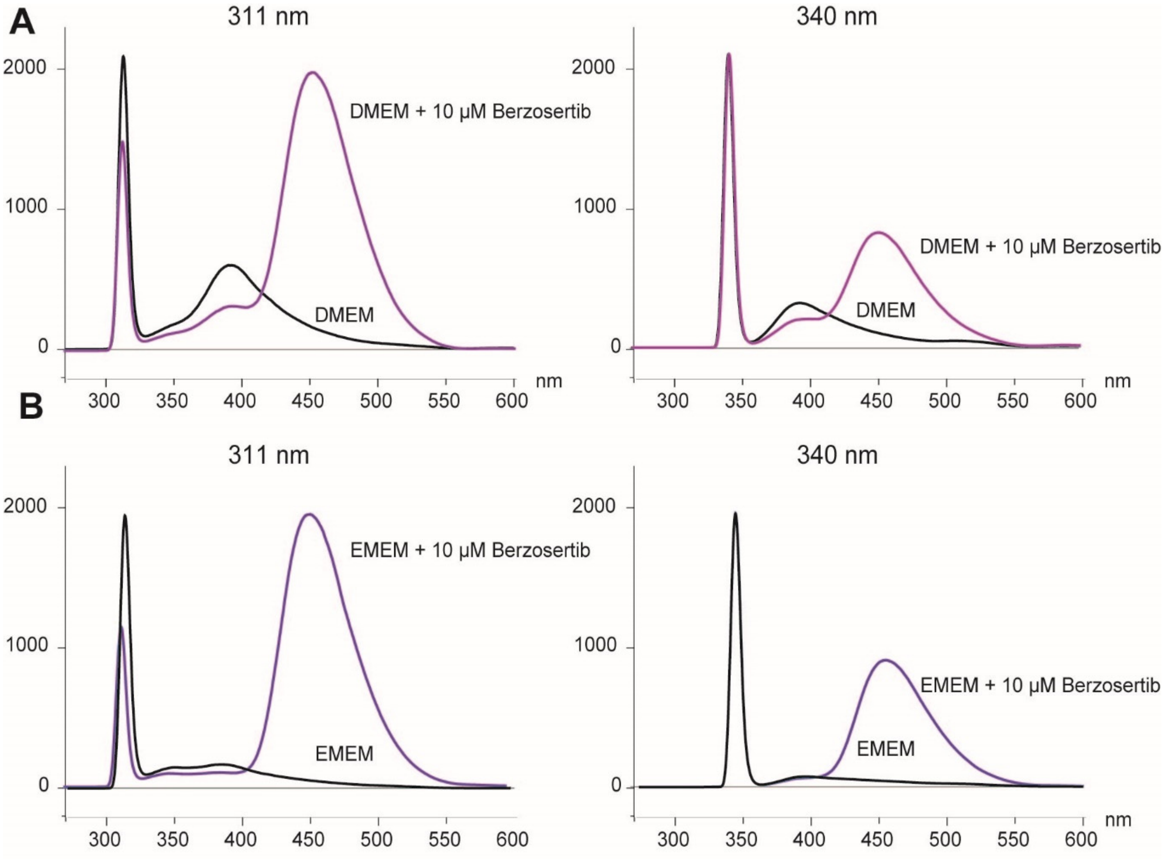

Supplementary Figure S3: Fluorescence Sprectrum of Berzosertib A Fluorescence spectrum of Berzosertib 10 µM in DMEM and DMEM alone at 311 nm and 340 nm. **B** Fluorescence spectrum of Berzosertib 10 µM in EMEM and EMEM alone 311 nm and 340 nm

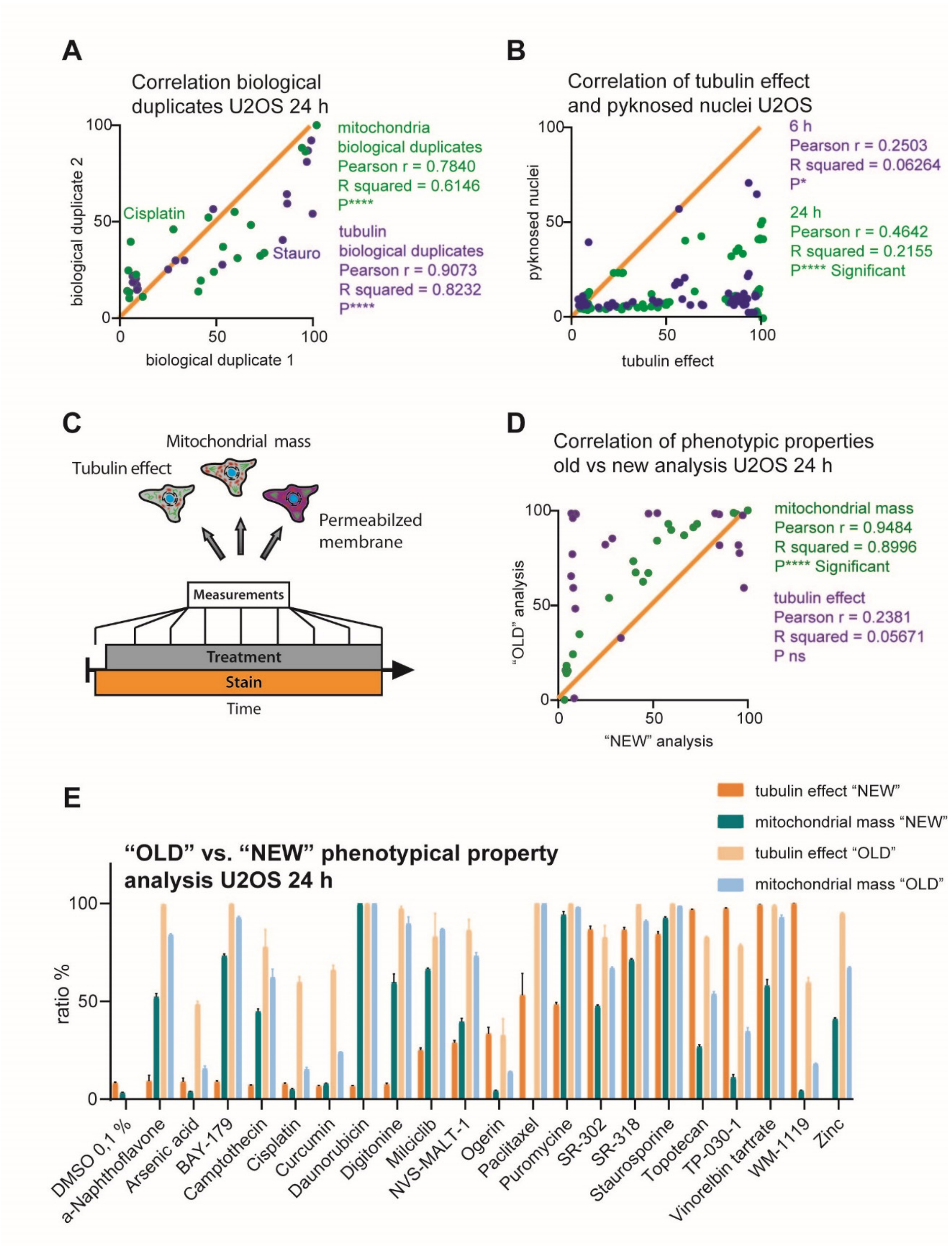

Supplementary Figure S4: Validation of Multiplex high Via protocol A Correlation of phenotypical analysis of tubulin mitochondrial mass increase of biological duplicates in U2OS cells after 24h. **B** Correlation of tubulin effect and pyknosed nuclei analysis in U2OS cells after 6

h (purple) and 24h (green). **C** General workflow of “old” analysis. **D** Correlation of “old” and “new” analysis of tubulin effect (purple) and mitochondrial mass (green) analysis in U2OS cells after 24h. **E** Cell count ratio of tubulin effect (orange) and mitochondrial mass increase (blue) of “old” and “new” analysis in U2OS cells after 24h of 10 µM of compound exposure (Suppl. tabl.3) in comparison to DMSO 0.1%. Error bars show SEM of technical triplicates.

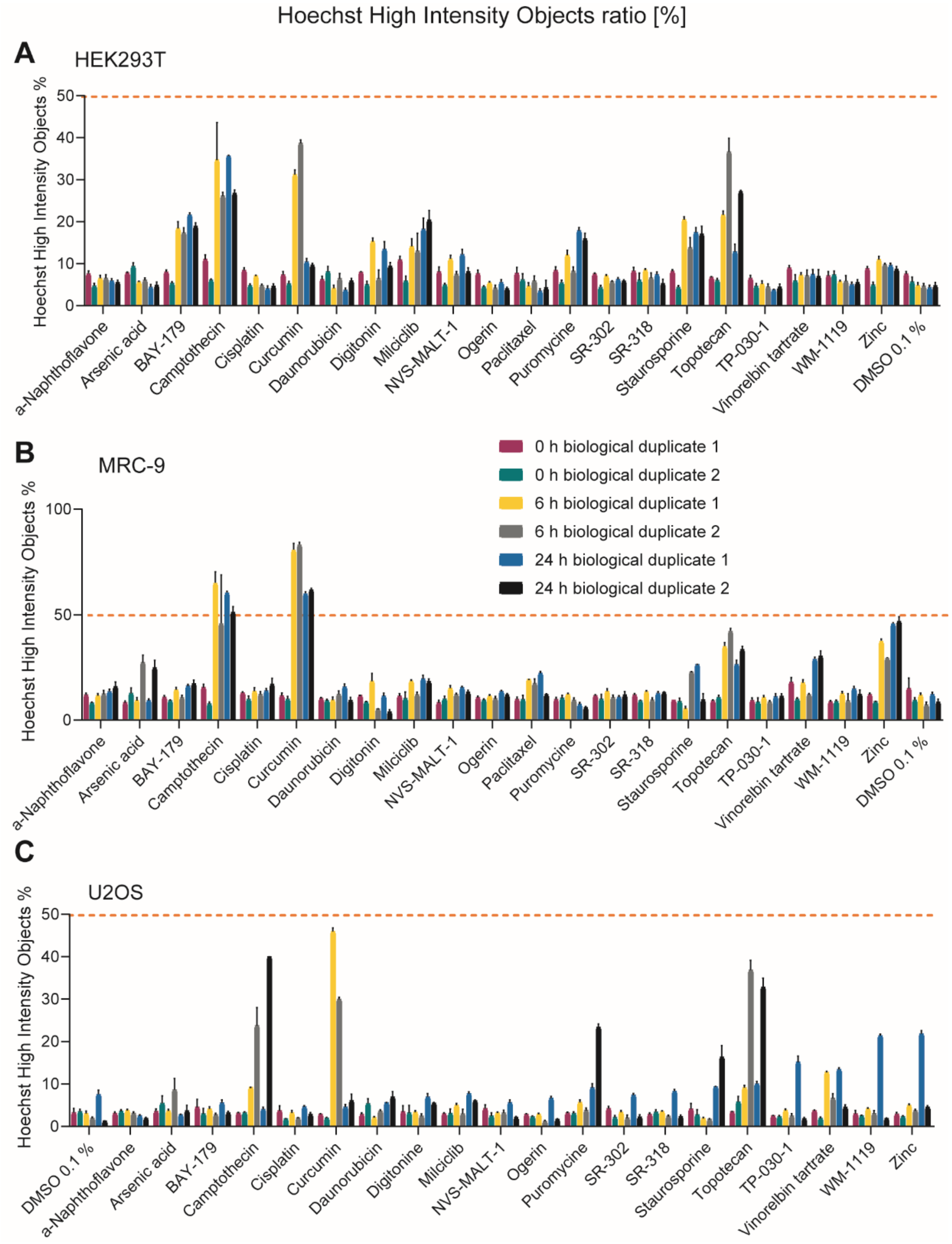

Supplementary Figure S5: Hoechst High Intensity Object Analysis. A Ratio of Hoechst High Intensity Objects after 0h, 6h and 24h of compound exposure (Supplementary Table S4) in HEK293T cells in comparison to DMSO 0.1% of biological duplicates. Error bars show SEM of technical triplicates. Property threshold at 50% marked red. **B** Ratio of Hoechst High Intensity Objects after 0h, 6h and 24h of compound exposure (Supplementary Table S4) in MRC-9 cells in

comparison to DMSO 0.1% of biological duplicates. Error bars show SEM of technical triplicates. Property threshold at 50% marked red. **C** Ratio of Hoechst High Intensity Objects after 0h, 6h and 24h of compound exposure (Supplementary Table S4) in MRC-9 cells in comparison to DMSO 0.1% of biological duplicates. Error bars show SEM of technical triplicates. Property threshold at 50% marked red.

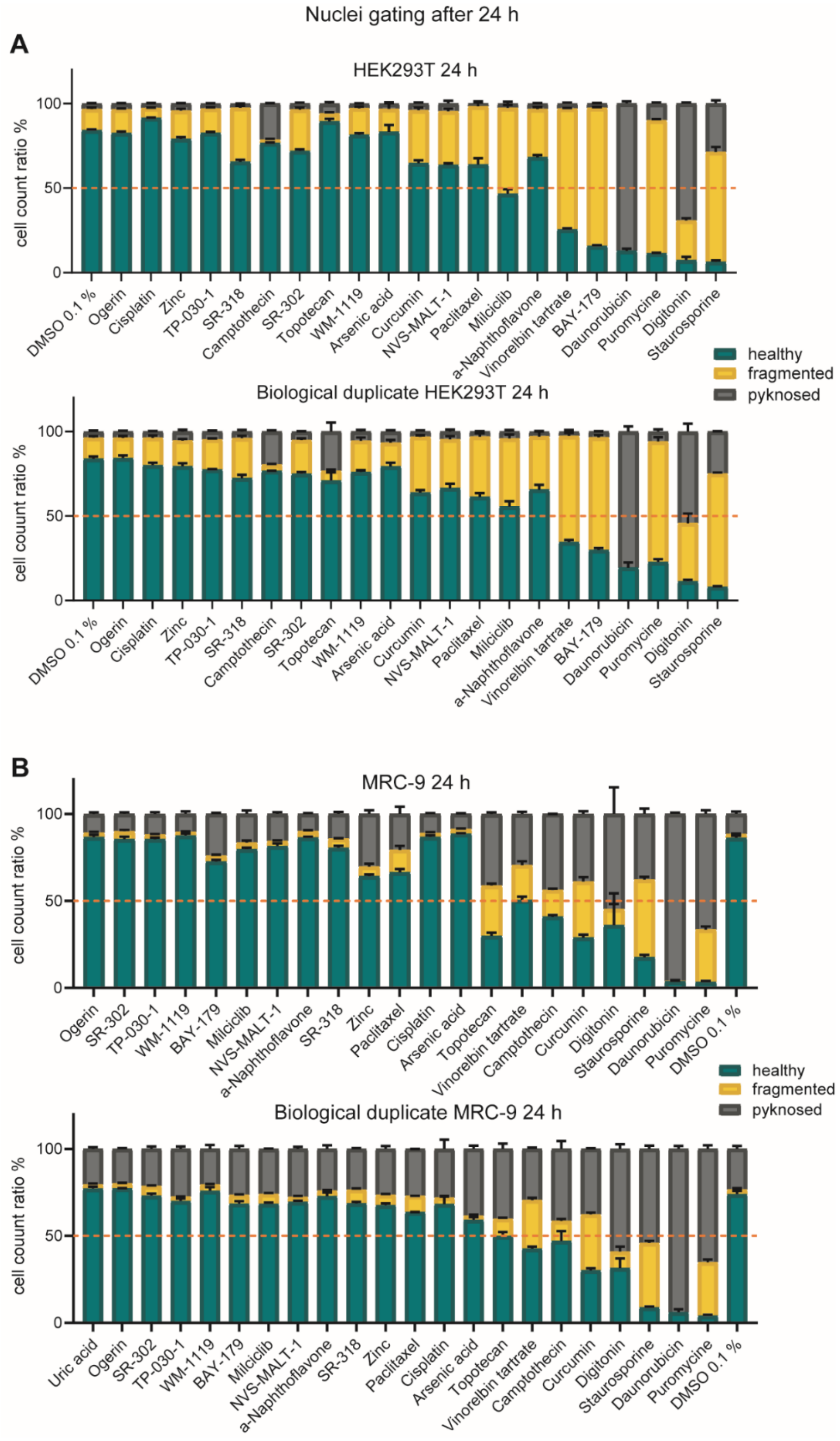

Supplementary Figure S6: Viability analysis over nuclei gating protocol A Cell count ratio of different Nuclei gating after 24h of 10 µM of compound exposure (Supplementary Table S4) in comparison to DMSO 0.1% in HEK293T cells. Error bars show SEM of technical triplicates.

Property threshold at 50% marked red. Both biological duplicates are shown. **B** Cell count ratio of different Nuclei gating after 24h of 10 µM of compound exposure (Supplementary Table S4) in comparison to DMSO 0.1% in MRC-9 cells. Error bars show SEM of technical triplicates. Property threshold at 50% marked red. Both biological duplicates are shown.

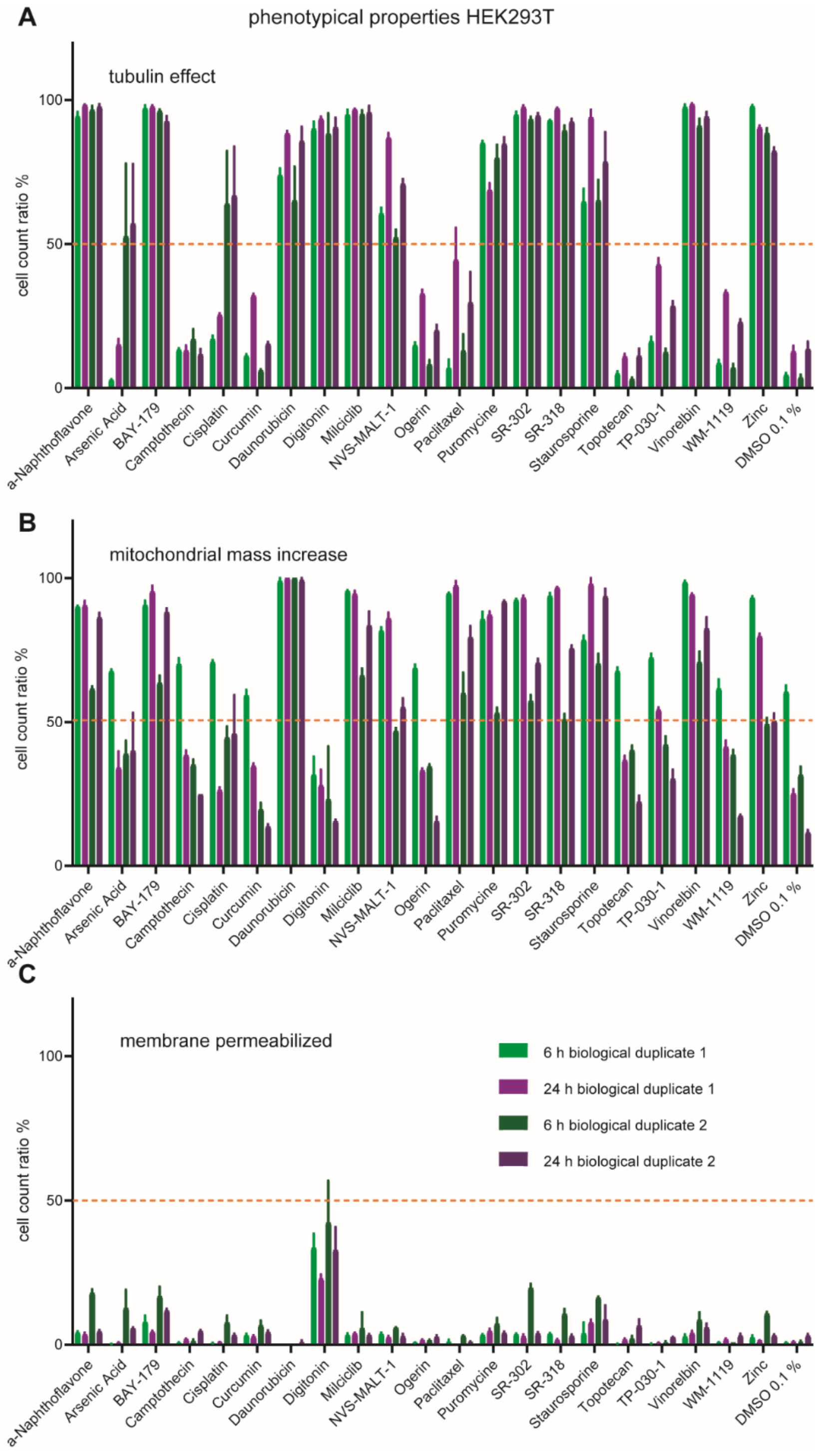

Supplementary Figure S7: Phenotypical property analysis in HEK293T cells. Cell count ratio of tubulin effect (S7 **A**), mitochondrial mass increase (S7 **B**) and membrane permeability (S7 **C**) of HEK293T cells after 6h and 24h of 10 µM of compound exposure (Supplementary Table S4)

in comparison to DMSO 0.1%. Error bars show SEM of technical triplicates. Property threshold at 50% marked in red. Both biological supplicates are shown.

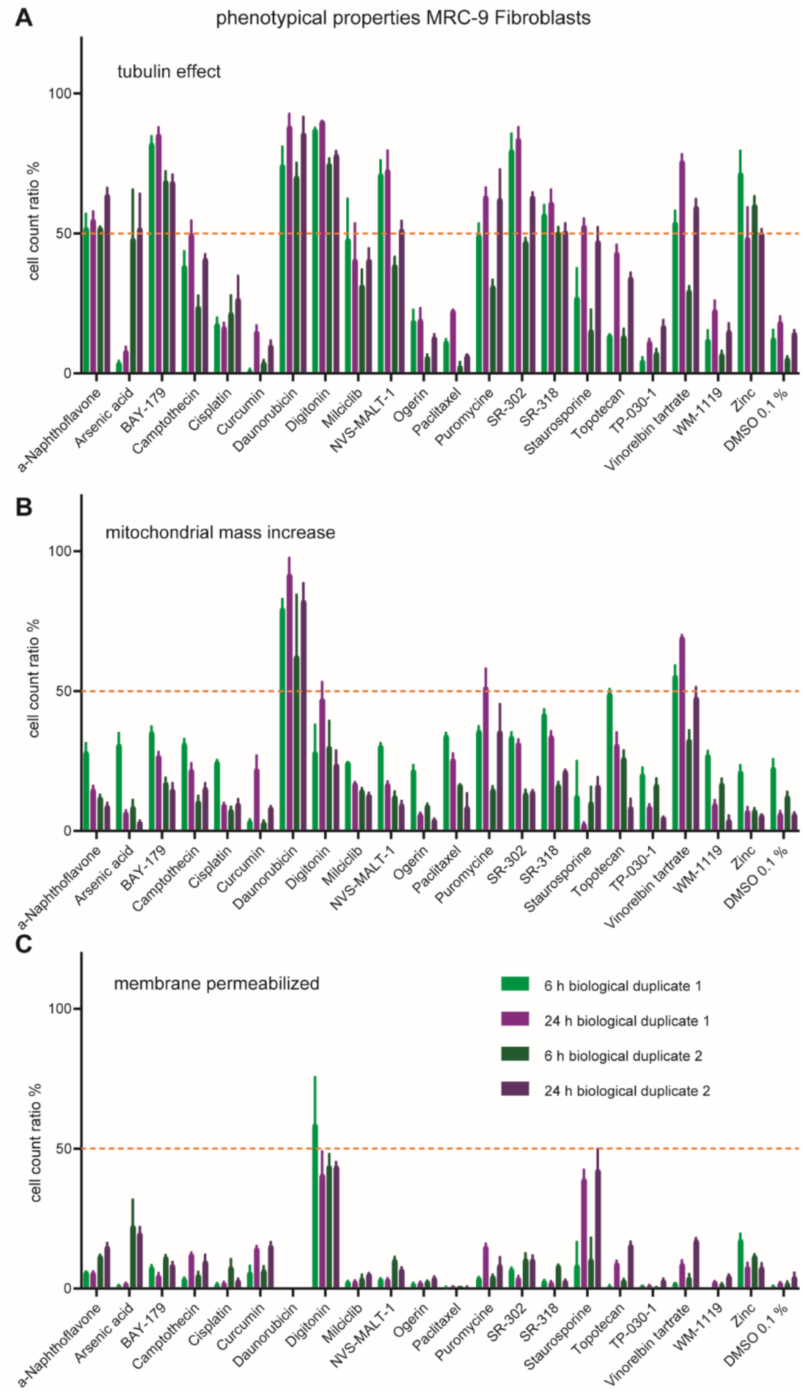

Supplementary Figure S8: Phenotypical property analysis in MRC-9 cells. Cell count ratio of tubulin effect (S7 **A**), mitochondrial mass increase (S7 **B**) and membrane permeability (S7 **C**) of MRC-9 cells after 6h and 24h of 10 µM of compound exposure (Supplementary Table S4) in comparison to DMSO 0.1%. Error bars show SEM of technical triplicates. Property threshold at 50% marked in red. Both biological supplicates are shown.

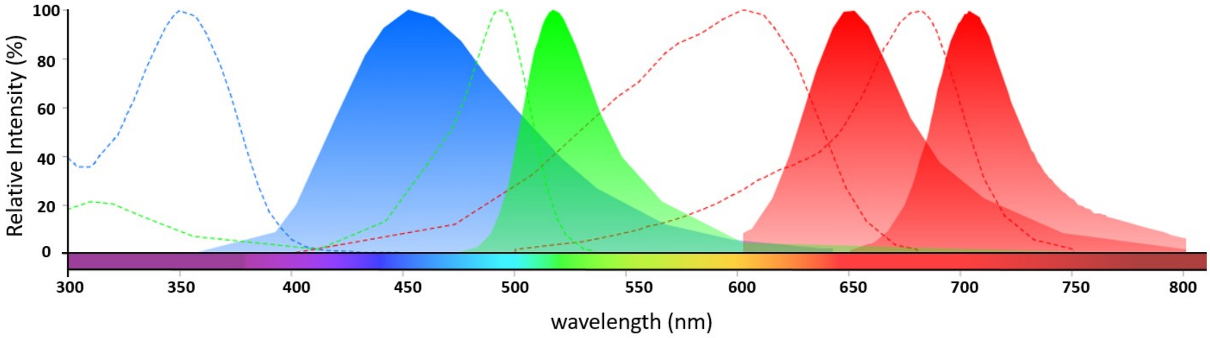

Supplementary Figure S9: Spectra Viewer visualization. Spectra Viewer visualization of fluorophore excitation and emission wavelengths (https://www.thermofisher.com/uk/en/home/life-science/cell-analysis/labeling-chemistry/fluorescence-spectraviewer.html). Hoechst (blue), BioTracker™ 488 Green Microtubule Cytoskeleton Dye (green), MitoTracker red (Red) and Annexin V Alexa Fluor 680 conjugate (deep red), as used in the protocol.

Supplementary Table S1: Concentrations in µM of tested cell staining dyes. Concentration used in described assays are marked in blue.

number	Hoechst33342	Yo-Pro-3	BioTracker™ 488 Green Microtubule Cytoskeleton Dye	Mitotracker™ red	Mitotracker™ far red
**1**	0.02	0.1	1	0.01	0.01
**2**	0.035	0.5	2	0.05	0.05
**3***	0.060	1	3	0.075	0.075
**4**	0.085	3.5	4	0.1	0.1
**5**	0.130	5	5	0.5	0.5
**6**	0.170	10	6	1	1

* concentration used in Multiplex and High Via Extend protocol

Supplementary Table S2: reference compounds tested in High-Via Extend protocol

Reference compound	Mode of action	Predominant cell death type	IC 50 14 h	IC 50 26 h	IC 50 42 h	IC 50 56 h
**digitonin**	detergent	lysis	10.6	11.6	11.5	15.1
**torin**	mTOR kinase inhibitor	Apoptosis, autophagy	0.8	0.8	0.7	0.7
**ricolinostat**	HDAC 6 inhibitor	Apoptosis, cell cycle arrest	n/a	13.5	13.4	13.7
**paclitaxel**	targets tubulin/no disassembly of mitotic spindle	Apoptosis, tubulin binder, cell cycle arrest	> 2	0.1	0.06	0.05
**staurosporine**	kinase inhibitor	Apoptosis	0.005	0.004	0.003	<0.001
**JQ1**	BET inhibitor	Apoptosis, cell cycle arrest	9.7	9.0	13.7	0.2
**berzosertib**	ATR/ATM-inhibitor	Apoptosis, DNA damage response	62.7	64.1	58.7	56.5
**milciclib**	CDK inhibitor	Apoptosis, cell cycle arrest	2.1	0.8	0.6	0.5
**camptothecin**	topoisomerase inhibitor	Apoptosis, DNA damage reposne	2.6	2.3	1.5	1.1

Supplementary Table S3: Compounds tested in FUCCI Assay System

**compound**	**mode of action**	**concentration [µM]**	**known cell cycle effect**	**main nuclei color**	**reference**
α-Naphtoflavone	flavone derivate, inhibitor of enzyme aromatase	10	G1 cell cycle arrest	red	[39,35]
Bromosporine	bromodomain (BET) inhibitor	10	increase of cells in G1 phase → cell cycle block	red	[74]
Camptothecin	topoisomerase inhibitor I	10	mitotic arrest	green	[27,75]
Daunorubicine	topoisomerase inhibitor II	10	DNA double strand breaks, cell cycle arrest	yellow	[47]
Doxorubicine	topoisomerase inhibitor II	10	G0/G1 cell cycle arrest after continuous treatment with 5 µM	yellow	[76]

HI-TOPK-032	TOPK inhibitor	10	TOPK serine/threonine kinase is phosphorylated during mitosis, G1 cell cycle arrest	yellow	[48]
JH-XI-05-01	SRPK1/2 inhibitor[1]	10	not described previously	red	[77]
Milciclib	CDK inhibitor	10	G1 cell cycle arrest	red	[37,38]
Mitoxantrone	topoisomerase inhibitor II	10	delay in cell cycle progression	green	[78,43,44]
Paclitaxel	targets tubulin/no disassembly of mitotic spindle	10	concentration-dependent G1 or Mitosis cell cycle arrest	red	[40,41]
Panobinostat	histone deacetylase (HDAC) inhibitor	10	G1/S cell cycle arrest	green	[79]
Puromycine	aminonucleoside antibiotic	10	effect on cell cycle checkpoints	red	[80]
Staurosporine	kinase inhibitor	10	dose-dependent cell cycle arrest in G1 or G2	green	[81]
T3-CLK	CLK inhibitor	10	G2/M cell cycle arrest	green	[82]

Supplementary Table S5: References used for Multiplex protocol

**compound**	**mode of action**	**concentration [µM]**
Ogerin	positive allosteric modulator of GPR68 (DCP probe)	10
TP-030-1	RIPK1 inhibitor (DCP probe)	10
WM-1119	KAT6A, KAT6B inhibitor (DCP probe)	10
SR-302	DDR1, DDR2, MAPK11, MAPK14 inhibitor (DCP probe)	10
NVS-MALT1	MALT1 allosteric inhibitor (DCP probe)	10
Zinc	trace element, corrosive	10
Cisplatin	interfering in DNA replication	10
Arsenic acid	toxic and corrosive chemical compound	10
SR-318	MAPK14 inhibitor (DCP probe)	10
α-Naphtoflavone	flavone derivate, inhibitor of enzyme aromatase	10
BAY-179	complex I inhibitor (DCP probe)	10
Curcumin	natural product	10
Milciclib	CDK inhibitor	10
Paclitaxel	targets tubulin/no disassembly of mitotic spindle	10
Topotecan	topoisomerase inhibitor	10
Digitonin	detergent	10
Camptothecin	topoisomerase inhibitor	10

Vinorelbine tartrate	vinca alkaloid, antimicrotubule agent	10
Staurosporine	kinase inhibitor	10
Puromycine	aminonucleosid antibiotic	10
Daunorubicine	anthracycline antibiotic, intercalate of DNA strands, ROS production	10

Supplementary Table S6: Trainings set. Compounds to train the machine learning algorithm for Multiplex protocol

reference compound	mode of action	predominant cell death type
**digitonin**	detergent	lysis
**paclitaxel**	tubulin binder, cell cycle arrest	apoptosis
**staurosporine**	kinase inhibitor	apoptosis
**milciclib**	CDK inhibitor	apoptosis, cell cycle arrest
**dmso**	solvent	healthy cells

Supplementary Table S7: Features of machine learning algorithm

Table.1: features used for machine learning algorithm in healthy/early apoptotic/late apoptotic/lysed and necrotic cells

cell region	feature*	cell region	feature*
Cellbody	Area	Nuclei	Area
Cellbody	Diameter	Nuclei	Diameter
Cellbody	Circumference	Nuclei	Circumference
Cellbody	Circularity	Nuclei	Circularity
Cellbody	Compactness	Nuclei	Compactness
Cellbody	Anisometry	Nuclei	Anisometry
Cellbody	mean intensity CH2	Nuclei	total intensity CH1
Cellbody	mean intensity CH3	Nuclei	mean intensity CH1
Cellbody	mean intensity CH4	Nuclei	mean intensity CH3

Cellbody	mean peak CH2	Nuclei	total peak CH3
Cellbody	mean peak CH4	Nuclei	total ridge CH1
Cellbody	mean hole CH2	Nuclei	mean ridge CH1
Cellbody	mean hole CH4	Nuclei	mean ridge CH3
Cellbody	mean ridge CH2		
Cellbody	mean valley CH2		
Cellbody	mean valley CH4		
Cellbody	mean edge CH4		
Cellbody	mean saddle CH2		
Cellbody	mean saddle CH4		

Table 2: features used for machine learning algorithm in healthy/pyknosed and fragmented cell nuclei

**cell region**	**feature***	**cell region**	**feature***
Nuclei	total intensity CH1	Nuclei	mean ridge CH1
Nuclei	total hole CH1	Nuclei	mean valley CH1
Nuclei	total valley CH1	Nuclei	mean edge CH1
Nuclei	total edge CH1	Nuclei	mean saddle CH1
Nuclei	total saddle CH1		
Nuclei	mean hole CH1		

Table S3: features used for machine learning algorithm in High Intensity Objects and Normal Intensity Objects based

**cell region**	**feature***	**cell region**	**feature***
Cellbody	mean intensity CH1	Nuclei	max intensity CH1
Cellbody	min intensity CH1	Nuclei	Nuc_cell_area<br>Nuc Area / Cellbody Area
Cellbody	max intensity CH1		

Table 4: features used for machine learning algorithm for Fucci assay in red/green and yellow nuclei

cell region	feature*	cell region	feature*
Cellbody	mean intensity CH3	Nuclei	total peak CH3
Cellbody	mean peak CH2	Nuclei	total edge CH2
Cellbody	mean peak CH3	Nuclei	total edge CH3
Cellbody	mean hole CH2	Nuclei	mean edge CH2
Cellbody	mean hole CH3		
Cellbody	mean ridge CH2		
Cellbody	mean ridge CH3		
Cellbody	mean valley CH2		
Cellbody	mean valley CH3		
Cellbody	mean saddle CH2		
Cellbody	mean saddle CH3		

Table 5: features used for machine learning algorithm in mitotic or apoptotic cells

cell region	feature*	cell region	feature*
Cellbody	total intensity CH4	Nuclei	total hole CH1
Cellbody	max intensity CH4	Nuclei	mean peak CH1
Cellbody	mean peak CH4	Nuclei	mean hole CH1
Cellbody	mean hole CH4	Nuclei	mean ridge CH1
Cellbody	mean ridge CH4	Nuclei	mean valley CH1
Cellbody	mean valley CH4		
Cellbody	mean saddle CH4		

Table 6: features used for machine learning algorithm in tubulin effect and no tubulin effect

cell region	feature*	cell region	feature*
Cellbody	total intensity CH2	Cellbody	mean peak CH2
Cellbody	mean intensity CH2	Cellbody	mean ridge CH2
Cellbody	max intensity CH2	Cellbody	mean edge CH2

Table 7: features used for machine learning algorithm in mitochondrial mass increased and mitochondrial mass normal

cell region	feature*	cell region	feature*
Cellbody	total intensity CH3	Cellbody	mean peak CH3
Cellbody	mean intensity CH3	Cellbody	mean hole CH3
Cellbody	max intensity CH3	Cellbody	mean ridge CH3
Cellbody	total peak CH3	Cellbody	mean valley CH3
Cellbody	mean saddle CH3		

Table 8: features used for machine learning algorithm in membrane permeabilized and membrane normal

cell region	feature*	cell region	feature*
Cellbody	total intensity CH2	Cellbody	mean hole CH2
Cellbody	total intensity CH4	Cellbody	mean hole CH3
Cellbody	mean intensity CH2	Cellbody	mean ridge CH2
Cellbody	mean intensity CH3	Cellbody	mean ridge CH3
Cellbody	max intensity CH2	Cellbody	mean ridge CH5
Cellbody	mean peak CH2	Cellbody	mean valley CH2
Cellbody	mean peak CH3	Cellbody	mean valley CH3
Cellbody	mean peak CH5	Nuclei	Compactness
Cellbody	mean edge CH2		

*

CH1: Hoechst33342 (DNA detection): Ex 405 nm/Em 447/60 nm, 500ms, 50%

CH2: BioTracker™ 488 Green Microtubule Cytoskeleton Dye (tubulin stain): Ex 488/Em 525/50 nm, 50 ms, 40%

CH3: MitoTracker red (mitochondrial mass detection): Ex 561 nm/Em 617/73 nm, 100 ms, 40%

CH4: Annexin V (apoptosis marker): Ex 640 nm/Em 685/40, 50 ms, 20%

CH5: bright field: 300ms, 100% transmission
